# Measurement of absolute copy number variation reveals association with essential hypertension

**DOI:** 10.1186/1755-8794-7-44

**Published:** 2014-07-15

**Authors:** Francine Z Marques, Priscilla R Prestes, Leonardo B Pinheiro, Katrina Scurrah, Kerry R Emslie, Maciej Tomaszewski, Stephen B Harrap, Fadi J Charchar

**Affiliations:** 1 Faculty of Science, Federation University Australia, Y Building, University Drive, Mt Helen, 3350, Ballarat, VIC, Australia; 2 National Measurement Institute, Sydney, NSW, Australia; 3 Department of Physiology, University of Melbourne, Melbourne, VIC, Australia; 4 Department of Cardiovascular Science, University of Leicester, Leicester, UK

**Keywords:** Copy number variation, Blood pressure, Hypertension, Extreme phenotypes, Droplet digital PCR

## Abstract

**Background:**

The role of copy number variation (CNV) has been poorly explored in essential hypertension in part due to technical difficulties in accurately assessing absolute numbers of DNA copies. Droplet digital PCR (ddPCR) provides a powerful new approach to CNV quantitation. The aim of our study was to investigate whether CNVs located in regions previously associated with blood pressure (BP) variation in genome-wide association studies (GWAS) were associated with essential hypertension by the use of ddPCR.

**Methods:**

Using a “power of extreme” approach, we quantified nucleic acids using ddPCR in white subjects from the Victorian Family Heart Study with extremely high (n = 96) and low (n = 92) SBP, providing power equivalent to 1714 subjects selected at random.

**Results:**

A deletion of the CNVs esv27061 and esv2757747 on chromosome 1p13.2 was significantly more prevalent in extreme high BP subjects after adjustment for age, body mass index and sex (12.6% vs. 2.2%; *P* = 0.013).

**Conclusions:**

Our data suggests that CNVs within regions identified in previous GWAS may play a role in human essential hypertension.

## Background

Essential hypertension is a multifactorial condition with a substantial contribution attributed to heritable genetic factors [1]. Unravelling the genetic predisposition to high blood pressure (BP), however, has proven challenging, mostly due to phenotypic heterogeneity [2]. Single nucleotide polymorphisms (SNPs) have been the most studied type of polymorphism in essential hypertension, particularly in genome-wide association (GWA) studies [2]. A meta-analysis of GWA studies in 200,000 individuals of European ancestry found that 29 SNPs at 28 loci were associated with changes in systolic blood pressure (SBP), diastolic blood pressure (DBP) and/or hypertension [3]. A majority of these SNPs, however, are not causal being mere proxies of genuinely functional alleles in the vicinity.

In contrast to SNPs, copy number variations (CNVs) are large polymorphisms such as insertions, deletions, translocations and inversions of genomic material varying from 1 kilobase to several megabases [4,5]. CNVs cover about 12% of the human genome [6] and importantly, half of them overlap with protein-coding regions [7]. In humans 85–95% of CNVs are associated with changes in expression of the affected genes [8]. There have been very few studies on the association between CNVs and essential hypertension [3,9]. One reason for this paucity of data is that most of the techniques available up to now, such as real-time quantitative PCR (qPCR) [10], have proven unreliable, as they do not measure the absolute number of copies, a prerequisite for meaningful analyses of association between CNVs and BP variation.

The combined use of limited dilutions, Poisson distribution and PCR was first described in 1992 [11], allowing the measurement of the absolute numbers of nucleic acid in a sample, later called digital PCR (dPCR) [12]. In this study, we took advantage of a new system for dPCR analysis, the droplet digital PCR (ddPCR) [13]. High precision in copy number measurements can be achieved due to the large number of droplets analysed (>10,000) for each sample [13]. The use of dPCR to measure CNVs is more reliable and precise compared to other techniques available [14,15].

The hypothesis of our study is that changes in genomic copy number may be associated with essential hypertension and/or BP variation but may have been missed in previous studies [9]. We first identified all the CNVs located in regions associated with BP/hypertension in the previous GWA meta-analysis. We then quantified these CNVs by high fidelity ddPCR to determine whether they are associated with hypertension. As a robust approach, we used the ‘power of extreme’ approach method [16,17] to enrich for rare but potent variants that could explain high BP.

## Methods

### Participants

The participants included in this study were selected from the Victorian Family Heart Study (VFHS), a healthy population-based cohort of European-descendants collected in Melbourne, Australia, to specifically study the family patterns in cardiovascular risk factors [18-21]. SBP was estimated based in the average of 2 lying SBP values and 2 standing SBP values. SBP and DBP for subjects on antihypertensive treatment (53.1% of the high BP group) were adjusted. Briefly, 10 mm Hg was added to systolic blood pressure and 5 mm Hg to diastolic blood pressure, as previously described [22,23]. In order to maximize the statistical power and enrich for rare variants in the current analysis, we selected a sex-matched sample of biologically unrelated subjects. We selected 96 and 92 subjects from the highest and lowest deciles for SBP in the VFHS, respectively. This was done in consideration of a mean SBP (122 mm Hg; SD: 14.3 mm Hg) in the VFHS, the mean SBPs of the high (166 mm Hg; SD: 12.3 mm Hg) and low (98 mm Hg; SD: 5.2 mm Hg) groups differed by ≈ 4.5 SDs. This provides power equivalent to 1714 subjects selected at random [22]. DNA was extracted from white blood cells. All subjects gave informed consent and the study was approved by the human research ethics committee at the Alfred Hospital, Melbourne, Australia.

In order to investigate whether having differential number of copies would lead to differential gene expression, we also genotyped forty Polish individuals of white European ancestry from the Silesian Renal Tissue Bank (SRTB), collected to study genetic aspects of human cardiovascular disease. DNA samples were extracted from white blood cells. Recruitment and phenotyping were as described [24,25]. Diagnosis of hypertension was as stipulated for the Silesian Hypertension Study. These subjects underwent elective unilateral nephrectomy because of non-invasive renal cancer. Samples from a pole of kidney unaffected by the neoplastic process had RNA extracted for gene expression analyses. All subjects gave informed consent and the study was approved by the human research ethics committee at the Medical University of Silesia, Poland.

### Selection of copy number variation polymorphisms

We evaluated all top genomic regions associated to blood pressure phenotypes in the meta-analysis of GWA studies by Ehret et al., [3] to see whether these regions contain CNVs defined by the Database for Genomic Variation (DGV, http://dgv.tcag.ca/dgv/app/home) [26], to which both HapMap Project and 1000 Genomes Project are linked (Additional file 1: Figure S1). This screening retrieved 4 locations with CNV loci (Table 1). Two of the locations contained overlapping CNVs: esv27061 and esv2757747, and dgv976e1, esv2656635, nsv908562 and dgv986e1 (Table 1).

**Table 1:** Single nucleotide polymorphisms associated with high blood pressure located in regions containing copy number variation (Build hg19, based on the Database for Genomic Variants and UCSC Genome Browser, search performed on 28 May 2014)

**SNP ID**	**CNV ID**	**Genomic landmark**	**Assay ID**
rs2932538	esv27061	chr1:112,692,629-113,246,263	Hs01327571
	esv2757747*	chr1:113,157,135-116,741,372	Hs01327571
rs7129220	nsv483076	chr11:10,193,294-10,352,897	Hs04399968
rs17608766	dgv976e1	chr17:44,083,914-45,277,333	Hs00313538
	esv2656635	chr17:44,281,452-45,168,501	Hs00313538
	nsv908562	chr17:44,828,931-45,102,413	Hs00313538
	dgv986e1	chr17:44,971,360-45,277,333	Hs00313538
rs1327235	dgv1306e1	chr20:10,892,138-11,116,725	Hs03126928

Footnote: SNP, single nucleotide polymorphism; ID, identification; CNV, copy number variation.

*essv21692 is described in the UCSC Genome Browser, however, it is a supporting structural variant as a single individual. Therefore, according to NCBI, essv21962 is a part of the CNV esv2757747.

### Droplet digital polymerase chain reaction (ddPCR)

This study followed the Minimum Information for Publication of Quantitative Digital PCR Experiments guidelines [27]. Four CNV assays were selected to genotype the 8 CNVs (Table 1). DNA samples, previously extracted from whole blood [18-21], were quantified by spectrophotometry. Given that one haploid human genome weighs 3.3 pg, and approximately 10,000 copies of genome per reaction are required for accurate ddPCR, we aimed to use 33 ng of DNA per reaction. Prior to PCR, DNA was restriction digested with either *XbaI*, *EcoRI* or *EcoRV* (Promega, Madison, Wisconsin, USA, 1 unit per 1 μg of DNA) for 1 hour at 37°C, followed by 20 minutes of heat inactivation at 65°C in a PCR thermal cycler (BioRad, Hercules, California, USA). PCR reactions were run in a total of 20 μl, containing 1 μl of the TaqMan assay (Life Technologies, Pleasanton, California, USA) specific for the CNV of interest, labelled with FAM dye, 1 μl of the copy number reference assay for the TaqMan® Copy Number Reference Assay RNaseP (87 bp, Life Technologies, Pleasanton, California, USA), labelled with VIC dye, and 10 μl of the ddPCR™ Supermix for Probes (BioRad, Hercules, California, USA). Cycling conditions were: initial 1 x 95°C for 10 min, followed by 45 cycles of denaturation for 15 sec (ramp rate at 2.5°C per sec), annealing-extension for 60 sec (ramp rate at 2.5°C per sec), and finished with a heat kill at 98°C for 10 min. Denaturation and annealing-extension temperature were optimised for each assay to improve distinction between positive and negative droplets (Additional file 1: Table S1). Droplets were generated in a QX100 droplet generator (BioRad, Hercules, California, USA). PCR was run in a C1000 Touch PCR thermal cycler (BioRad, Hercules, California, USA), and the results were read in a QX100 droplet reader (BioRad, Hercules, California, USA). In order to confirm the genotypes, 29 to 67% (depending on the assay) of the samples were randomly repeated at least twice in independent experiments.

### Data analyses

ddPCR data were analysed using the QuantaSoft software version 1.3.1.0, which measured the fraction of positive droplets and calculated the amount of template per droplet based on a Poisson distribution, which precision estimates a 95% confidence interval (CI) for each droplet. Agreement with normal distribution was assessed by histograms and estimation of skewness and kurtosis tests. A logistic regression adjusting for age, BMI and sex was performed to test the association between the CNVs and the extreme high and low BP groups. Independent-sample (two-tailed) Wilcoxon tests were used to identify if losses or gains of CNVs were associated with SBP or DBP in each group. Due to the small sample size, we did not adjust Wilcoxon tests by age, sex or BMI. The CNVs nsv483076, dgv976e1, esv2656635, nsv908562 and dgv986e1 were mostly monomorphic, so no statistical analyses were performed. Differences with *P* < 0.05 were considered statistically significant. Statistical package SPSS for Windows (Release 17.0, 2008) was used in the statistical analysis.

## Results

Mean SBP, DBP, age and BMI values were significantly higher in extreme high when compared to low BP groups (Table 2) [22]. The mean number of droplets analysed (±standard error of measurement) for all the samples ranged between 12729 ± 98 and 14725 ± 122 droplets, depending on the assay. Samples in which number of copies did not fall within the 95% Poisson CI for absolute number of 1, 2 or 3 copies per diploid genome, and samples that failed to amplify were repeated at least twice with consistent results, and therefore were excluded from further analyses. One sample was excluded for CNVs esv27061 and esv2757747 (0.5%), 7 for CNV nsv483076 (3.7%), 2 for the CNVs dgv976e1, esv2656635, nsv908562 and dgv986e1 (1%) and 14 for CNV dgv1306e1 (7.4%). We observed between 5466 ± 244 and 7872 ± 369 copies of DNA, depending on the assay. There was a high concordance between replicates of the same sample (data not shown). Additional file 1: Figure S2 shows an example of the quality of the copy calls, reporting replicates with either 1, 2 or 3 copies.

**Table 2:** Characteristics of the extreme low and high blood pressure subjects from the Victorian family heart study

**Characteristic**	**Extreme low BP (n = 92)**	**Extreme high BP (n = 96)**	***P*-value**
SBP	98.6 ± 5.2 mmHg	165.9 ± 12.3 mmHg	*P* < 0.001
DBP	64.3 ± 7.2 mmHg	94.4 ± 10.8 mmHg	*P* < 0.001
Age	32.6 ± 14.5 years old	55.1 ± 8.3 years old	*P <* 0.001
BMI	22.7 ± 3.2 kg.m^-2^	28.3 ± 4.5 kg.m^-2^	*P* < 0.001
Males (%)	47 (51.1%)	48 (50%)	*P* = 0.88

Footnote: BP, blood pressure; SBP, systolic blood pressure; DBP, diastolic blood pressure; BMI, body mass index.

Values are represented are average ± standard deviation.

**Figure 1:**
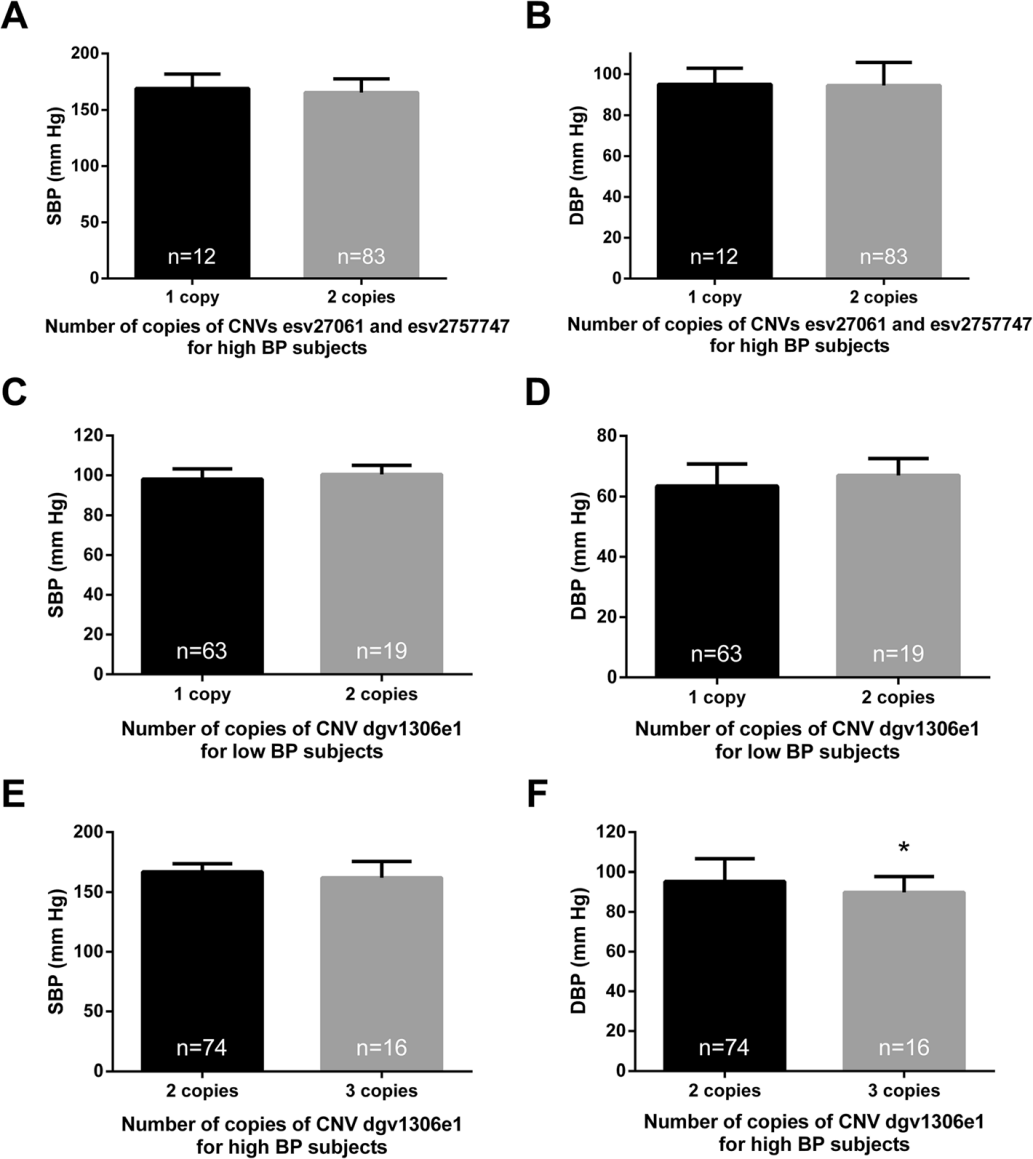
**Systolic blood pressure (SBP) and diastolic blood pressure (DBP) according to the genotype of CNVs.** According to the genotype for the CNVs esv27061 and esv2757747, there was no statistically significant difference in **(A)** SBP and **(B)** DBP in the extreme high blood pressure (BP) group. According to the genotype for the CNV dgv1306e1, there was no difference in **(C)** SBP and **(D)** DBP in the extreme low blood pressure group, while **(E)** there was a significant decrease in DBP in the extreme high BP group (* indicates *P* = 0.024), and **(F)** no change in SBP. Independent-sample Wilcoxon tests were performed between subjects with losses or gains in copy number in each BP group. Graphs represent mean, error bars represent standard deviation.

There was a significantly higher prevalence of a deletion in the overlapping CNVs esv27061 and esv2757747 on chromosome 1 in individuals from extreme high BP (n = 12) than those from extreme low BP (n = 2) group (12.6% vs. 2.2%, respectively; 95% confidence interval: 0.005–0.53; β = 0.05; *P* = 0.013) after adjustment for BMI, age and sex (Table 3 and Additional file 1: Figure S3). Within the stratum of high extreme of SBP, there were no significant difference in either SBP (+3.65 mmHg, *P* = 0.884) or DBP (+0.53 mmHg, *P* = 0.263) between subjects with 1 or 2 copies of these CNVs (Figures 1A and B). In the extreme low BP group the increase was of greater magnitude (+6.03 mmHg SBP and 7.84 mmHg DBP), but the number of subjects with the deletion was very small to perform statistical analyses (n = 2).

**Table 3:** Copy number variation (CNV) frequency in the extreme low and high cohort

	CNV esv27061/esv2757747*		**CNV nsv483076**		**CNV dgv976e1/esv2656635/nsv908562/dgv986e1**	**CNV dgv1306e1**	
**Number of copies**	**1 copy (%)**	**2 copies (%)**	**2 copies (%)**	**3 copies (%)**	**2 copies (%)**	**2 copies (%)**	**3 copies (%)**
Low BP: sample size (%)	2 (2.2)	90 (97.8)	90 (100)	0	91 (100)	63 (76.8)	19 (23.2)
High BP: sample size (%)	12 (12.6)	83 (87.4)	88 (98.9)	1 (1.1)	93 (100)	74 (82.2)	16 (17.8)

*P = 0.013 after adjustment for age, body mass index and sex.

Footnote: CNV, copy number variation; BP, blood pressure.

Although there was no association between CNV dgv1306e1 on chromosome 20p12.2 and the extreme high and low BP (Additional file 1: Table S2), subjects from the extreme low BP group with 3 copies had higher, but non-significant, SBP (+2.4 mmHg, *P* = 0.069) and DBP (+3.5 mmHg, *P* = 0.066) (Figure 1C and D). Subjects from extreme high BP group with 3 copies of CNV dgv1306e1 had no significant difference in SBP (-4.5 mmHg, *P* = 0.308) than those with 2 copies, but they had significantly lower DBP (-5.5 mmHg, *P* = 0.024) (Figure 1E and F).

No association was observed between the phenotypes investigated and the CNV nsv483076 on chromosome 11p15.4, in which only one subject of the extreme high BP group presented a duplication (Table 3).

The frequencies of the CNVs found in this study were compared to the DGV data (Additional file 1: Table S2). We observed that the presence of CNVs in the studies described in the DGV was different from some of the ones found in the present study. There was no gain in number of copies in the CNVs esv27061 and esv2757747 for all participants we studied, while the reported prevalence of gain in the DGV is 5.8% and 0.8%, respectively (Additional file 1: Table S2). For the CNV dgv1306e1, we observed a higher prevalence of variation of 20.3%, while the DGV reports only no variance.

All subjects genotyped for the SRTB cohort had 2 copies of the CNVs esv27061 and esv2757747, and therefore, we could not investigate if there was a correlation between gene expression and the deletion of a copy number in this region (data not shown).

## Discussion

This study is the first, to our knowledge, to investigate copy number variation in regions previously associated with BP in genome-wide association studies by the use of absolute DNA quantitation of droplet digital PCR and the ‘power of extreme’ approach. Significantly higher frequency of a deletion of the overlapping CNVs esv27061 and esv2757747 on chromosome 1 was observed in those with extreme high BP compared to low BP subjects. These CNVs are overlapping with the SNP rs2932538, which was previously associated with hypertension [3]. This indicates that CNVs in this region could contribute to the previous positive association in the GWA meta-analysis. Although no association was observed between the other three regions studied, subjects from extreme high SBP with 3 copies of the CNV dgv1306e1 in chromosome 20 had significantly lower DBP. Surprisingly, the frequency of copy number in our cohort of 184 European-descendent subjects was very different than the one in the DGV, which suggests that the data available in this database might be biased due to less reliable measurement techniques.

Although we reported here that a deletion of the CNVs esv27061 and esv2757747 was more prevalent in hypertensive subjects, we could not find a significant association with BP. This is likely a consequence of the sampling design employed in this study. The selection of subjects with extreme high and extreme low from a population-based sample such as the VFHS offers power in terms of the comparisons between, but not necessarily within the extreme groups. Factors such as low prevalence of certain variants and high standard deviation in the extreme high group militate against detection of effects within groups. Nevertheless, the observed BP differences with the deletion of the CNVs esv27061 and esv2757747 were of consistent direction in both high and low extreme groups. Also our data is consistent with the GWA meta-analysis, in which the loci containing SNP rs2932538 and CNVs esv27061/esv2757747 on chromosome 1 was associated with hypertension, but had a small effect (β = 0.049) [3], very comparable to our result (β = 0.05). Other parallels between our CNV data and the GWA meta-analysis were observed for the CNV dgv1306e1, which showed a stronger association between the SNP rs1327235 in chromosome 20 (where the CNV dgv1306e1 is located) and DBP (*P* = 1.4 × 10^-15^) than SBP (*P* = 1.9 × 10^-8^) or hypertension (*P* = 4.6 × 10^-4^) [3].

The CNV esv2757747, located on 1p13.2-1p13.1, is approximately 3.5 megabase pairs long (Mb) and encompasses 45 coding genes, including the one previously associated with BP coding moloney leukemia virus 10 (*MOV10*) [3], and several genes for long non-coding RNAs (lncRNAs) of unknown function, but expressed in relevant tissues such as heart and kidney (Additional file 1: Table S3). LncRNAs, important for epigenetic regulation, are emerging as a new class of non-coding RNAs possibly involved in cardiovascular disease [28]. Amongst the genes in this region is the one coding for the nerve growth factor (*NGF*). This gene been previously implicated in BP regulation in the spontaneously hypertensive rats (SHR), the most studied model of hypertension [29,30].

The esv27061 variant is a smaller CNV of 553,635 bp on chromosome 1p13.2, which encompass 9 genes, including *MOV10,* and a gene for a microRNA, miR-4256 (Additional file 1: Table S3). Besides *MOV10*, none of them have been previously associated with BP or hypertension. The CNV dgv1306e1, of 224,588 bp on 20p12.2, initially contained the jagged 1 gene (*JAG1*) when the meta-analysis was published [3]. In the newest human genome browser, GRCh37/hg19, *JAG1* no longer maps within CNV dgv1306e1, while one new transcript (*C20orf187*) and several new lncRNAs, with unknown roles but transcribed in relevant tissues to BP regulation such as kidney, have been described in this region (Additional file 1: Table S3).

The combined usage of comparative genomic hybridisation (CGH) arrays and qPCR identified 16 CNVs associated with BP distributed across 8 chromosomes in the SHR [31]. The expression of several genes within theses CNVs were validated in kidney, heart and spleen [31]. Of particular interest was the gene coding for EGL nine homolog 1 (*Egln1*), which is located in an orthologous region in humans and was shown in a large F_2_ cross to be associated with BP [31]. No validation, however, has been done in humans so far. A study which sequenced the whole-genome of the SHR identified 588 CNVs overlapping with 688 genes [32], but gene expression was not investigated. There are few studies aimed at finding CNVs that could contribute to the pathophysiology of essential hypertension in humans. The first GWA study for CNVs in essential hypertension, which designed their own arrays using the Agilent CGH platform, analysed ~2,000 hypertensive subjects with British ancestry and ~3,000 controls, and found no association between any CNVs and hypertension [9]. Interestingly, from the initial 11,541 loci targeted, only 3,432 CNVs (31.6%) passed quality control filters indicating the difficulty in accurate quantitation. Another study which used a similar population size converted data from SNP-arrays to CNVs based on allele intensity, and found 7 CNVs associated with hypertension [33]. None of these regions, however, are the same ones identified in this study or in the GWA meta-analysis [3].

In order to find the functional role for the CNVs associated with hypertension in this study, we genotyped 40 Polish subjects with normal or high BP, for which we have matching kidney samples. All subjects, however, had 2 copies of the CNVs esv27061 and esv2757747, and therefore, we could not investigate if there was a correlation between gene expression and the deletion of a copy number in this region (data not shown). The lack of variability in this population may be explained by the different genetic background of the two cohorts (white-Australian vs. Polish), the small sample size of the Polish cohort analysed, or by the enrichment for rare variants in the extreme BP groups [34].

The frequency of loss or gain of copy number described in the DGV (UCSC Genome Browser and GRCh37/hg19 assembly, search performed on 29 May 2014) is presented in Additional file 1: Table S2 [4,6,35]. For the CNVs esv27061 and esv2757747, we did not observe any gain in copy number for all subjects genotyped. Furthermore, the higher prevalence of the CNV dgv1306e1 in our cohort (20.3%) compared to the DGV (0%) could be a result of the enrichment for rare variants by the use of the ‘power of extreme’ approach for high and low BP used in the present study [34], or high diversity between different populations for this and CNV dgv1306e1 loci. The studies that reported these first frequencies, however, used CGH arrays and small sample sizes (n = 40, [4] n = 55 [4] and n = 270 [6]) compared to more recent whole-genome studies. CGH arrays are based on hybridisation of labelled DNA to genomic clones, together with the hybridisation of a reference sample; therefore it is a relative measurement. Literature suggests <70% reproducibility in replicate experiments between platforms and algorithms for CGH and SNP arrays used for CNV detection [36]. The 1000 Genomes Project, which used a combination of exome sequencing and low-coverage whole-genome [37], will hopefully be able to rectify the frequency of CNVs reported in databases.

Technical difficulties and inaccuracy have been reported in the measurement of CNVs by qPCR [10]. For single CNV investigations, studies comparing qPCR and dPCR have proven the superiority of dPCR in accuracy and reproducibility [14,15]. One of the reasons is the number of replicates necessary for accurate qPCR results. For example, at least 8 replicates are needed when using qPCR to detect ratios of relative quantity greater than 1.25 with 95% power [15]. Some samples were excluded because an absolute 1, 2 or 3 copy number (0.5-7.4% depending on the assay) was not obtained. This may be caused by mosaicism in the population, in accordance with recent findings by another group using ddPCR [38], or degradation of this region in some DNA copies.

We have to acknowledge several limitations of the present study. One limitation relates to the absence of a clear agreement regarding quality control for results obtained by the new technology of ddPCR. The technique is labour intensive and each sample needs to be analysed individually. The small sample sizes would normally limit power for detection of genetic association. However, the use of the extreme phenotype approach enhances the power to detect association [16,17,22,39,40]. Previous calculations [39] of our effective sample size estimated power to be equivalent to an analysis of 1714 unselected subjects [20]. One consideration about the use of extreme phenotypes, however, is that it could select for rare syndromes and associated diseases, therefore decreasing the physiological relevance of the associations. To minimise this possible issue, subjects with other forms of hypertension than essential hypertension were excluded increasing the genetic power of our analyses. Finally, we did not validate the findings in a larger population because these regions have been previously associated with BP and hypertension in over 200,000 subjects [3].

## Conclusions

Our results show an association between the CNVs esv27061 and esv2757747 and extreme high BP subjects, and the CNV dgv1306e1 and DBP in hypertensive subjects by the use of the recently available and highly precise ddPCR in an extreme phenotype study. The results are in accordance to largest GWA meta-analysis, and together they suggest that CNVs in at least one of these regions could be an underlying factor to the initial association. The function of these CNVs in BP regulation, however, needs to be clarified.

## Abbreviations

BMI: Body mass index; BP: Blood pressure; CNV: Copy number variation; DBP: Diastolic blood pressure; ddPCR: Droplet digital polymerase chain reaction; DGV: Database for Genomic Variation; DNA: Deoxyribonucleic acid; GWA: Genome-wide association; PCR: Polymerase chain reaction; qPCR: Real-time polymerase chain reaction; RNA: Ribonucleic acid; SBP: Systolic blood pressure; SNP: Single nucleotide polymorphism; SRTB: Silesian Renal Tissue Bank; VFHS: Victorian Family Heart Study.

## Competing interests

The authors have declared no conflict of interest.

## Authors’ contributions

FZM participated in the study design, carried out the molecular genetic studies and statistical analyses, and drafted most of the manuscript. PRP participated in the ddPCR experiments. LBP and KRE participated in the design of the ddPCR experiments. KS participated in the design of the study and supervised statistical analyses. MT participated in the design of the study. SBH and FJC conceived the study, participated in its design and coordination. All authors helped draft the manuscript and read and approved the final version.

## Pre-publication history

The pre-publication history for this paper can be accessed here:

http://www.biomedcentral.com/1755-8794/7/44/prepub

## Supplementary Material

Additional file 1 Online Data Supplement. Click here for file
