# Supplementary material for: Measurement of absolute copy number variation reveals association with essential hypertension
Source: BMC Med Genomics. 2014 Jul 15;7:44. doi: 10.1186/1755-8794-7-44 (PMC4107748; doi:10.1186/1755-8794-7-44)
Supplement: Additional file 1 — Online Data Supplement. [file 1755-8794-7-44-S1.docx]

Online Data Supplement for

**Measurement of absolute copy number variation reveals association with essential hypertension**

**Short title**: Copy number variation in essential hypertension

Francine Z Marques^1^ Priscilla R Prestes,^1^ Leonardo B Pinheiro,^2^ Katrina Scurrah,^3^ Kerry R Emslie,^2^ Maciej Tomaszewski,^4^ Stephen B Harrap,^3^ Fadi J Charchar^1*^

^1^School of Health Sciences, Federation University Australia, VIC, Australia

^2^National Measurement Institute, Lindfield, NSW, Australia

^3^Department of Physiology, University of Melbourne, VIC, Australia

^4^Department of Cardiovascular Science, University of Leicester, UK

*Correspondence to Prof Fadi Charchar, Y Building, University Drive, University of Ballarat, Mt Helen, 3350. P: (03) 5327 6098, Fax: (03) 5327 9602

Email: f.charchar@ballarat.edu.au

**Table S1.** Single nucleotide polymorphisms associated with high blood pressure located in regions containing copy number variation, and the experimental conditions used for the droplet digital PCR (Build hg19, based on the Database for Genomic Variants and UCSC Genome Browser, search performed on 28 May 2014).

| SNP ID | Genomic landmark of SNP | CNV ID | Genomic landmark of CNV | Assay ID | Annealing temperature | Denaturation temperature | Amplicon size |
| --- | --- | --- | --- | --- | --- | --- | --- |
| rs2932538 | chr1:113,216,543 | esv27061 | chr1:112,692,629-113,246,263 | Hs01327571 | 60^o^C | 96^o^C | 79 bp |
|  |  | esv2757747* | chr1:113,157,135-116,741,372 | Hs01327571 | 60^o^C | 96^o^C | 79 bp |
| rs7129220 | chr11:10,350,538 | nsv483076 | chr11:10,193,294-10,352,897 | Hs04399968 | 60^o^C | 95^o^C | 96 bp |
| rs17608766 | chr17:45,013,271 | dgv976e1 | chr17:44,083,914-45,277,333 | Hs00313538 | 60^o^C | 94.4^o^C | 105 bp |
|  |  | esv2656635 | chr17:44,281,452-45,168,501 | Hs00313538 | 60^o^C | 94.4^o^C | 105 bp |
|  |  | nsv908562 | chr17:44,828,931-45,102,413 | Hs00313538 | 60^o^C | 94.4^o^C | 105 bp |
|  |  | dgv986e1 | chr17:44,971,360-45,277,333 | Hs00313538 | 60^o^C | 94.4^o^C | 105 bp |
| rs1327235 | chr20:10,969,030 | dgv1306e1 | chr20:10,892,138-11,116,725 | Hs03126928 | 62^o^C | 95^o^C | 76 bp |

Footnote: SNP, single nucleotide polymorphism; ID, identification; CNV, copy number variation.

* essv21692 is described in the UCSC Genome Browser, however, it is a supporting structural variant as a single individual. Therefore, according to NCBI, essv21962 is a part of the CNV esv2757747.

**Table S2.** Presence of loss or gain of copy number variation in studies described in the Database for Genomic Variants (DGV, based on the (Build hg19, based on the Database for Genomic Variants and UCSC Genome Browser, search performed on 28 May 2014) compared to all samples analysed in this study.

| CNV ID | Our study* (%) | DGV* (%) |
| --- | --- | --- |
| esv27061 | Losses: 14/187 (7.5%) | Losses: 3/451 (0.7%)[[1](#_ENREF_1)] |
|  | Gains: 0/187 (0%) | Gains: 26/451 (5.8%) |
| esv2757747 | Losses: 14/187 (7.5%) | Losses: 1/270 (0.4%)[[2](#_ENREF_2)] |
|  | Gains: 0/187 (0%) | Gains: 2/270 (0.8%) |
| nsv483076 | Losses: 0/179 (0%) | Losses: 1/39 (2.6%)[[3](#_ENREF_3)] |
|  | Gains: 1/179 (0.6%) | Gains: 1/39 (2.6%) |
| dgv976e1 | 0/184 (0%) | 0/270 (0%) [[2](#_ENREF_2)]** |
| esv2656635 | 0/184 (0%) | Loss: 7/1151 (0.6%)[[4](#_ENREF_4)] |
| nsv908562 | Gains: 0/184 (0%) | Gains: 1/6533 (0.0001%) [[5](#_ENREF_5)] |
| dgv986e1 | 0/184 (0%) | 0/270 (0%) [[2](#_ENREF_2)] |
| dgv1306e1 | Gains: 35/172 (20.3%) | Gains: 0/270 (0%)[[2](#_ENREF_2)]*** |

Footnote: CNV ID: copy number variation identification; DGV; Database for Genomic Variants.

*Calculated as the total number of subjects with loss or gain of a copy over the total number of subjects studied.

**Until April 2013, 207 of 270 (77%) were described as having gain of copy number for this CNV.

***Until April 2013, 2 of 39 (5%) were described as having changes in copy number for this CNV.

**Table S3.** Genes and non-coding RNA located in the region of the copy number variations (CNVs) esv27061, esv2757747 and dgv1306e1 (search on 29 May 2014 using UCSC Genome Browser and GRCh37/hg19 assembly).

| CNV | Size (bp) | Coding genes and lncRNA located in CNV region |
| --- | --- | --- |
| esv27061 | 553,635 | **Coding genes:** AX747733, *CAPZA1, CTTNBP2NL, DKFZp547A023, MOV10, RHOC, SnoU13, ST7L, WNT2B*  **microRNAs:** hsa-mir-4256 |
| esv2757747 | 3,584,238 | **Coding genes:** *AKR7A2P1, AMPD1, AP4B1, AX747733*, *BCAS2, BCL2L15, BC048113, BC036361, BC023568, BC047723, BX648855, BC037540, CAPZA1, CASQ2, CSDE1, DCLRE1B, DENND2C, FAM19A3, HIPK1, HP08777, LOC100287722, LOC643441, LRIG2, MAB21L3, MAGI3, MOV10, NGF, NHLH2, NRAS, OLFML3, PHTF1, PPM1J, PTPN22, RHOC, RSBN1, SIKE1, SLC16A1, SLC22A15, ST7L, SYCP1, SYT6, TRIM33, TSHB, TSPAN2, VANGL1*  **LncRNAs:** TCONS_00000491, TCONS_000 504, TCONS_000 95, TCONS_00001631, TCONS_00000274, TCONS_00000275, TCONS_00000096, TCONS_00001109, TCONS_00000279, TCONS_0000280, TCONS_00001113, TCONS_00001634, TCONS_00000052, TCONS_00001115, TCONS_00000282 |
| dgv1306e1 | 224,588 | No coding genesLncRNAs: TCONS_00028108, TCONS_00028500, TCONS_00028501 |

Footnote: CNV, copy number variation; bp, base pairs; lncRNA, long non-coding RNAs.

**
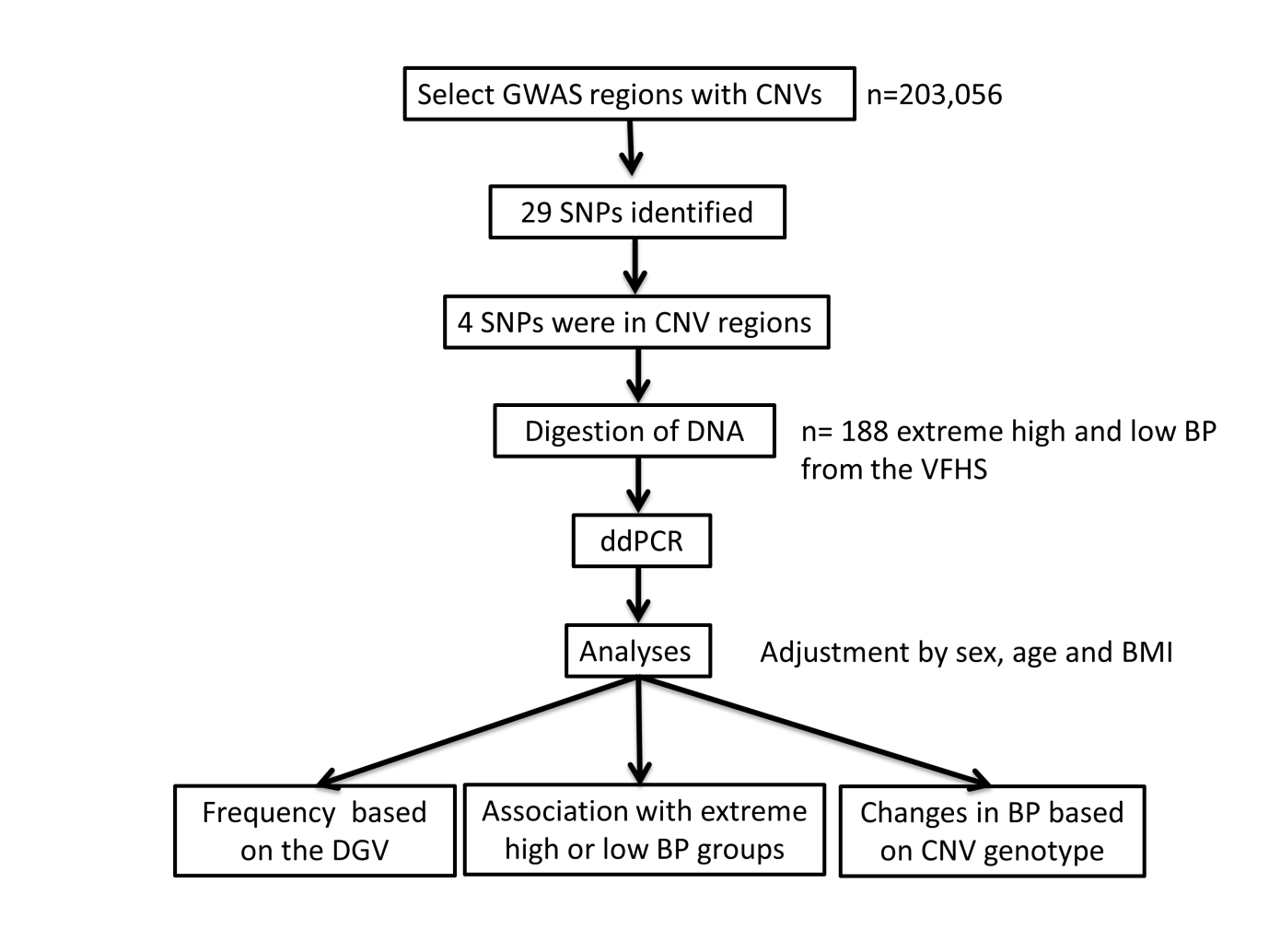
Figure S1.** Flowchart representing the selection of the copy number variation polymorphisms investigated in this study. Legend: GWAS, genome-wide association study; CNVs, copy number variation; SNPs, single nucleotide polymorphisms; VFHS, Victorian Family Heart Study; DGV, Database for Genomic Variants; BP, blood pressure.

**
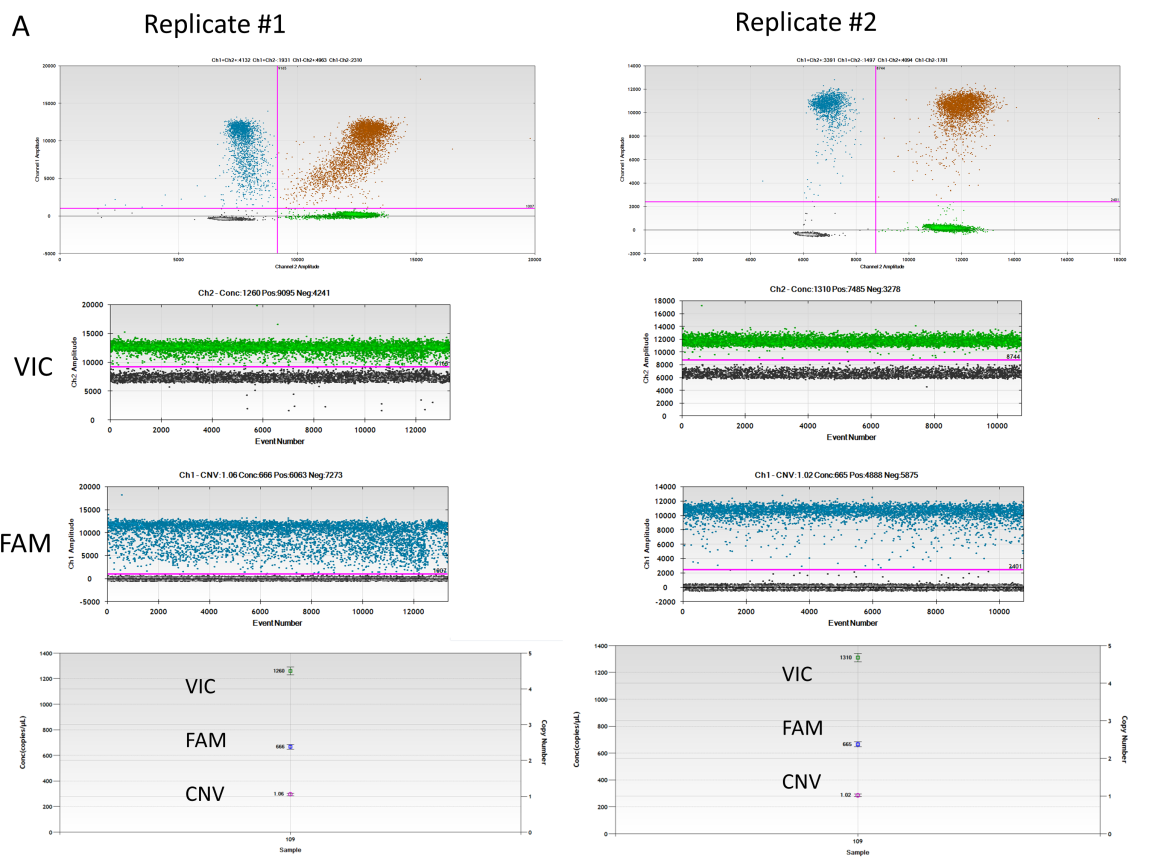
**

**
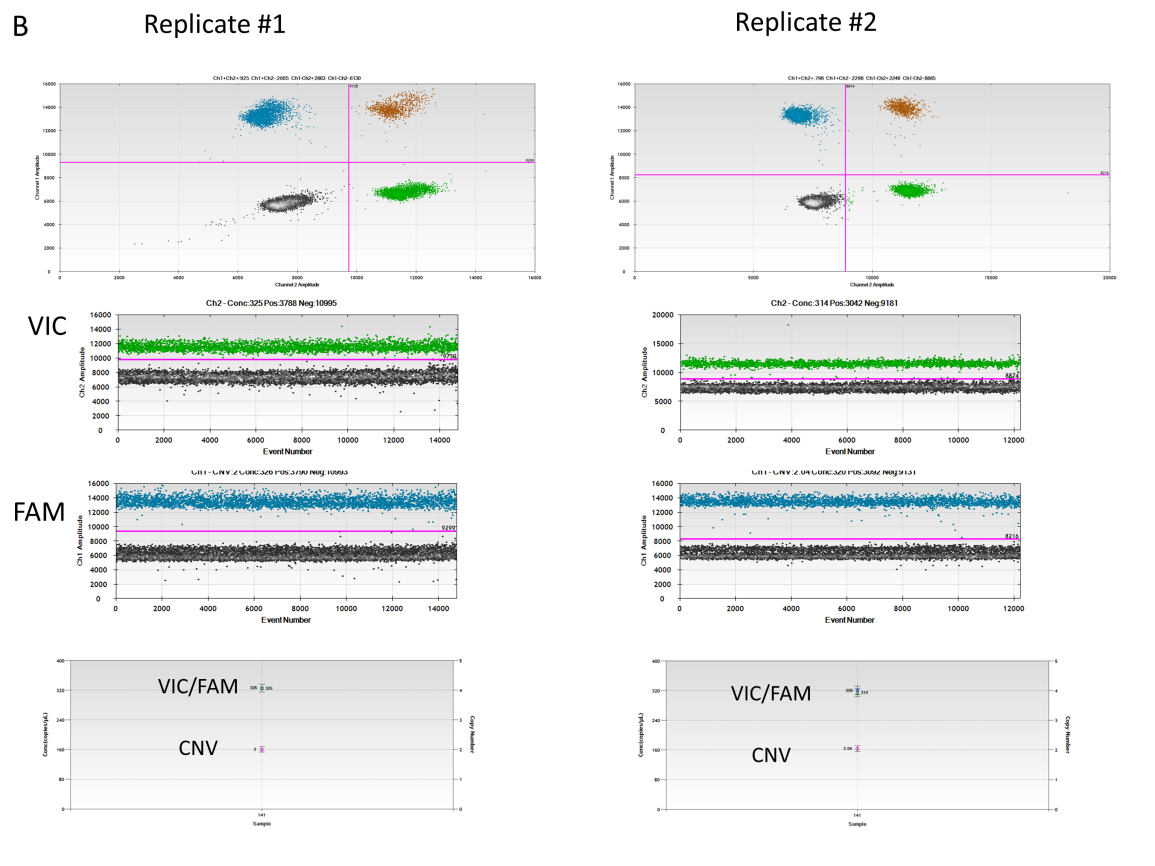
**

**
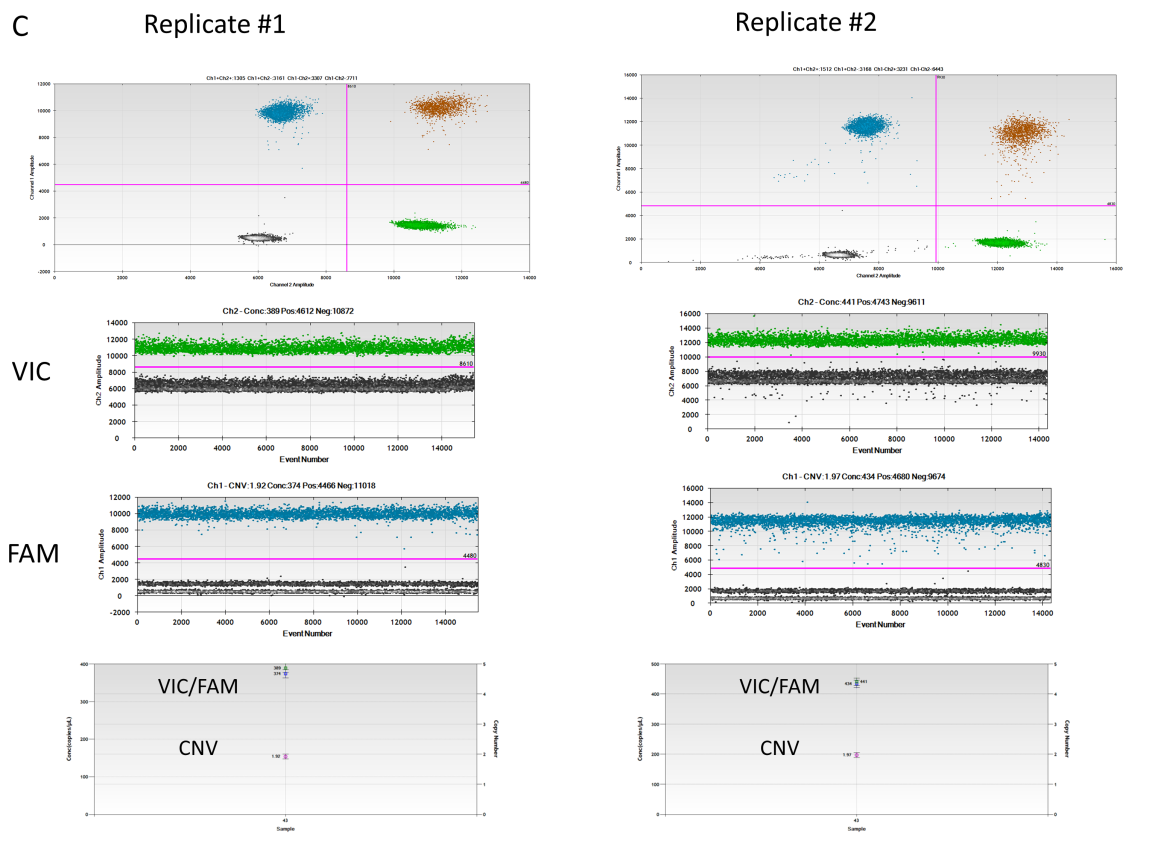
**

**
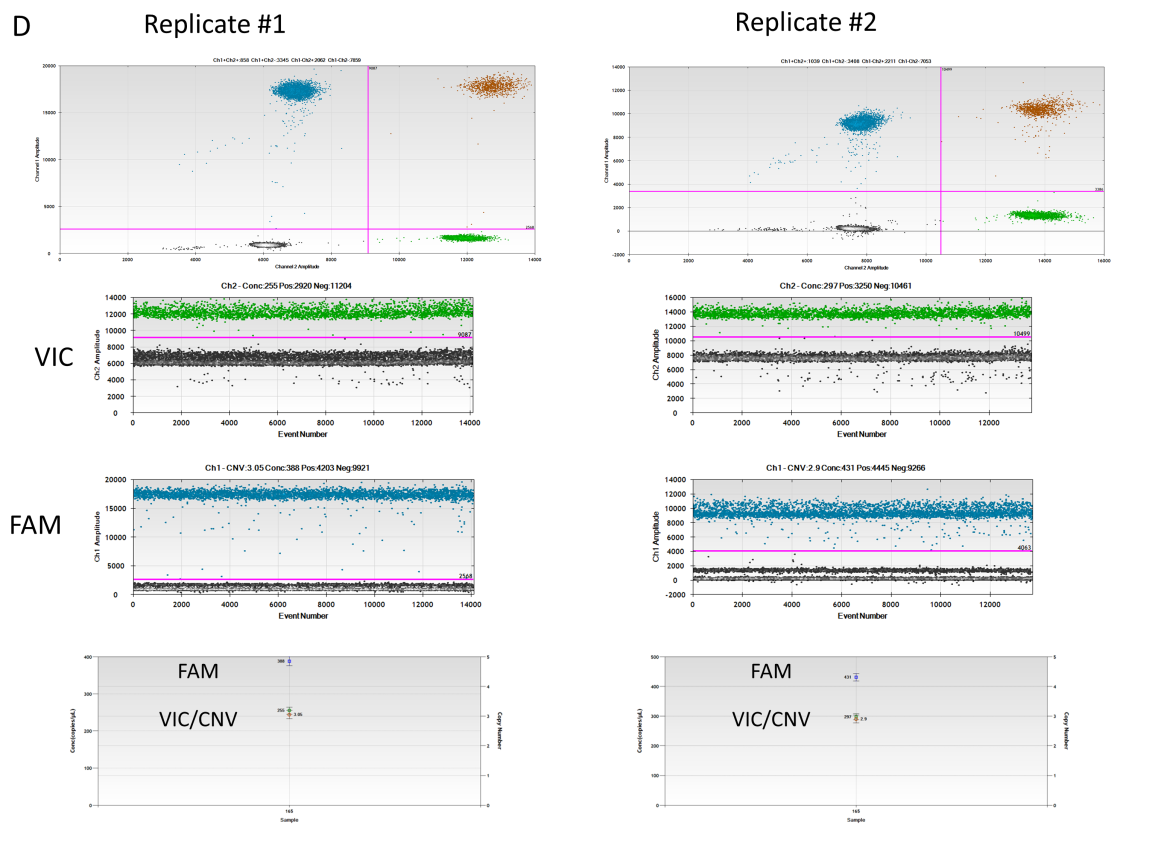
**

**Figure S2.** Results from droplet digital PCR (ddPCR). (A) Example of independent replicates of ddPCR showing one copy of the CNVs esv27061 and esv2757747. Top graph is a scatter plot graph of FAM (Y axis) vs VIC (X axis). Second and third graphs show the amplitude of positive droplets for VIC (green) and FAM (blue), respectively. The last graph shows the concentration (in copies/ul) of VIC and FAM, and the calculated copy number. (B) Example of independent replicates of ddPCR showing two copies of the CNV nsv483076. (C) Example of independent replicates of ddPCR showing two copies of the CNVs dgv976e1, esv2656635, nsv908562 and dgv986e1. (D) Example of independent replicates of ddPCR showing three copies of the CNV dgv1306e1.



 **Figure S3.** Frequency of the copy number variations (CNVs) esv27061 and esv2757747 in the extreme low and high blood pressure (BP) groups. A deletion of the CNVs esv27061 and esv2757747 is significantly more prevalent in the extreme high BP group (n=95) than in the extreme low BP subjects (n=92). Graphs represent frequency of the genotype in the sample, * indicates *P*=0.013.

**References**

1. Conrad DF, Pinto D, Redon R, Feuk L, Gokcumen O, Zhang Y, Aerts J, Andrews TD, Barnes C, Campbell P *et al*: **Origins and functional impact of copy number variation in the human genome**. *Nature* 2010, **464**(7289):704-712.

2. Redon R, Ishikawa S, Fitch KR, Feuk L, Perry GH, Andrews TD, Fiegler H, Shapero MH, Carson AR, Chen W *et al*: **Global variation in copy number in the human genome**. *Nature* 2006, **444**(7118):444-454.

3. Iafrate AJ, Feuk L, Rivera MN, Listewnik ML, Donahoe PK, Qi Y, Scherer SW, Lee C: **Detection of large-scale variation in the human genome**. *Nat Genet* 2004, **36**(9):949-951.

4. Abecasis GR, Auton A, Brooks LD, DePristo MA, Durbin RM, Handsaker RE, Kang HM, Marth GT, McVean GA: **An integrated map of genetic variation from 1,092 human genomes**. *Nature* 2012, **491**(7422):56-65.

5. Xu H, Poh WT, Sim X, Ong RT, Suo C, Tay WT, Khor CC, Seielstad M, Liu J, Aung T *et al*: **SgD-CNV, a database for common and rare copy number variants in three Asian populations**. *Hum Mutat* 2011, **32**(12):1341-1349.
